# The Multidisciplinary Approach to Alzheimer's Disease and Dementia. A Narrative Review of Non-Pharmacological Treatment

**DOI:** 10.3389/fneur.2018.01058

**Published:** 2018-12-13

**Authors:** Chiara Zucchella, Elena Sinforiani, Stefano Tamburin, Angela Federico, Elisa Mantovani, Sara Bernini, Roberto Casale, Michelangelo Bartolo

**Affiliations:** ^1^Neurology Unit, University Hospital of Verona, Verona, Italy; ^2^Alzheimer's Disease Assessment Unit, Laboratory of Neuropsychology, IRCCS Mondino Foundation, Pavia, Italy; ^3^Department of Neurosciences, Biomedicine and Movement Sciences, University of Verona, Verona, Italy; ^4^Neurorehabilitation Unit, Department of Rehabilitation, HABILITA, Bergamo, Italy

**Keywords:** Alzheimer's disease, dementia, non-pharmacological intervention, rehabilitation, caregiver, interprofessional team

## Abstract

**Background:** Alzheimer's disease (AD) and dementia are chronic diseases with progressive deterioration of cognition, function, and behavior leading to severe disability and death. The prevalence of AD and dementia is constantly increasing because of the progressive aging of the population. These conditions represent a considerable challenge to patients, their family and caregivers, and the health system, because of the considerable need for resources allocation. There is no disease modifying intervention for AD and dementia, and the symptomatic pharmacological treatments has limited efficacy and considerable side effects. Non-pharmacological treatment (NPT), which includes a wide range of approaches and techniques, may play a role in the treatment of AD and dementia.

**Aim:** To review, with a narrative approach, current evidence on main NPTs for AD and dementia.

**Methods:** PubMed and the Cochrane database of systematic reviews were searched for studies written in English and published from 2000 to 2018. The bibliography of the main articles was checked to detect other relevant papers.

**Results:** The role of NPT has been largely explored in AD and dementia. The main NPT types, which were reviewed here, include exercise and motor rehabilitation, cognitive rehabilitation, NPT for behavioral and psychological symptoms of dementia, occupational therapy, psychological therapy, complementary and alternative medicine, and new technologies, including information and communication technologies, assistive technology and domotics, virtual reality, gaming, and telemedicine. We also summarized the role of NPT to address caregivers' burden.

**Conclusions:** Although NPT is often applied in the multidisciplinary approach to AD and dementia, supporting evidence for their use is still preliminary. Some studies showed statistically significant effect of NPT on some outcomes, but their clinical significance is uncertain. Well-designed randomized controlled trials with innovative designs are needed to explore the efficacy of NPT in AD and dementia. Further studies are required to offer robust neurobiological grounds for the effect of NPT, and to examine its cost-efficacy profile in patients with dementia.

## Introduction

Dementia represents one of the major health problems in elderly individuals, with progressive deterioration of cognition, daily activity functioning and behavior that together lead to disability. Worldwide, approximately 40 million people over 65 years suffer from dementia, and 70% of them are affected by Alzheimer's disease (AD) that represents the most diffuse type of dementia. The incidence of dementia increases with age, which is the most important risk factor. Therefore, the prevalence of AD is expected to increase in the future, in parallel with the progressive aging of the population. In the European Union, the most reliable estimates forecast dementia cases to overcome 15 million in 2020 (1–3). Moreover, the improved life conditions in developing countries will lead to increased life expectancy, and therefore to higher incidence of dementia (4).

No disease-modifying treatment has been demonstrated to be effective in AD. Cholinesterase inhibitors and memantine, which are the only commercially available symptomatic drugs, may improve cognitive and behavioral outcomes, but their clinical impact remains modest and controversial (5–7). Despite research has been focused on the early [i.e., mild cognitive impairment (MCI) with biological evidence of AD] or preclinical stages of the disease, and new disease-modifying treatments have been tested to reduce the supposed pathophysiological changes of AD (i.e., amyloid deposition), no significant clinical effect has been achieved, to date (8).

Because of the limited efficacy of current pharmacological therapy and with the knowledge that caring for people with AD and dementia requires the involvement of a large number of professionals (e.g., psychologists, occupational therapists, etc.) and caregivers to offer a comprehensive and individualized management, research focused on non-pharmacological treatment (NPT). NPT may improve function, independence, and quality of life (QoL) in agreement with the International Classification of Functioning, Disability and Health (ICF) (9), is non-invasive, safe and has few side effects (10). NPT encompasses a wide range of interventions to improve patients' symptoms, reduce the stress of caregivers, and ameliorate the environment. NPT is based on different methodologies, ranging from simpler (e.g., environmental interventions) to complex approaches (e.g., virtual reality, home automation) (11). These interventions are not aimed to influence the underlying pathophysiological mechanisms but to maintain function and participation as long as possible, as the disease progresses, thus reducing disability and improving patient's and caregiver's QoL. However, a direct effect of NPT on cognition through neural plasticity/adaptation cannot be excluded in early disease stages.

The present narrative review summarized evidence on NPTs for AD and dementia. Because of the large bulk of data on this topic, we focused on the main NPT strategies. We did not report and discuss data on NPT in MCI, an intermediate state between intact cognition and dementia (12), because of the large number of studies that would have required a paper apart.

## Search Strategy

PubMed and the Cochrane database of systematic reviews were searched for studies published from 2000 to 2018 with a search string including the following terms: Alzheimer's disease, dementia, behavioral and psychological symptoms, non-pharmacological, intervention, therapy, exercise, motor, rehabilitation, neurorehabilitation, cognitive, training, stimulation, occupational, psychological, psychotherapy, multicomponent, multidimensional, complementary and alternative medicine, aromatherapy, music therapy, art therapy, massage and touch, technology, information and communication, assistive device, domotics, virtual reality, gaming, serious games, telemedicine, related terms, and MeSH. Only papers written in English were considered. The bibliography of the main articles (reviews, systematic reviews, meta-analyses) was also checked to detect other relevant papers.

In consideration of the great heterogeneity of interventions that are classified as NPT and of the variety of outcome measures reported in the different studies, this work was not conceived as a systematic review of the literature, but as a narrative one to provide a useful tool for clinicians on the state of the art of NPT in dementia.

## Exercise and Motor Rehabilitation

A growing amount of evidence suggests that a healthy lifestyle can reduce the risk of cardiovascular diseases (13, 14), osteoporosis, diabetes, and depression (15). A number of prospective cohort studies suggested that a regular physical activity may enhance cognitive function and reduce the risk of AD and other dementias and delay their onset or progression (16–24). A recent paper derived from the population-based Mayo Clinic study of aging on 280 MCI patients showed that moderate intensity midlife physical activity was significantly associated with a decreased risk of incident dementia (25). Regular physical exercise is recommended to all older adults, and indeed to those at increased risk of AD, as it may improve physical health, reduce frailty, lower the risk of depression, improve cognitive function in the short and long term (26–31).

Conversely, the evidence on the protecting role of short-term, single-component physical activity interventions seems to be largely insufficient (32).

Exercise has been documented to improve physical health and well-being (33), to reduce behavioral and psychological symptoms of dementia (BPSD) (34–36), and to enhance performance in the activities of daily living (ADL) (37, 38) in patients with AD or dementia.

A Cochrane review including 17 randomized controlled trials (RCTs) confirmed the positive effect of exercise programs in reducing the progression of dependence in ADL in people with dementia but showed limited evidence of benefit for the remaining outcomes (i.e., cognition, BPSD, QoL, depression, mortality, caregiver burden, and use of healthcare services) (39). This finding is of some relevance because preserving functional independence in ADL is critical for improving the QoL of people with dementia and their caregivers and delaying institutionalization.

The exercise interventions explored in dementia include a variety of training methods (e.g., aerobic exercise, resistance training or weightlifting, balance, and flexibility training), and the physical/motor outcome measures were quite different across studies (e.g., chair stand tests, timed up and go, timed walking tests). Comparative studies to explore the most effective exercise intervention, the amount of exercise, the most sensitive and significant outcome, and the role of physical activity in different patients' groups are needed.

According to animal models, a number of mechanisms may mediate the effects of exercise on brain and cognition (40). It has been suggested that exercise may improve vascular health by reducing blood pressure (41, 42), arterial stiffness (42), oxidative stress (43), systemic inflammation (44), and enhance endothelial dysfunction (45), all of which are associated with better cerebral perfusion (46, 47). Exercise may also preserve neurons and promote neurogenesis, synaptogenesis, (48–50), and neuronal plasticity (51–55).

Imaging studies in aging people confirmed the positive association between physical activity and gray matter volume. Both aerobic fitness and coordinative exercises was associated with reduced brain atrophy in the frontal and temporal regions and the hippocampus, all areas that are critical for higher cognitive function (56–58). A recent review on the association between fitness, physical activity and gray matter volume showed that both cross-sectional studies and randomized interventions consistently suggested a change in the size of prefrontal cortex and hippocampus after moderate intensity exercise in elderly people (59).

Neurotrophins, in particular the brain-derived neurotrophic factor, may be the common pathway of these mechanisms, may mediate the exercise-driven brain responses and can drive neuronal plasticity, improve cognition, and protect neurons against insult (60–63).

Future studies aimed to identify lifestyle, behavioral and/or biologic factors that may increase the likelihood of successful brain aging would help implementing preventive intervention to reduce age-related brain atrophy and consequently the risk for cognitive decline.

Conversely, the effect of physical exercise on other biological measures of AD pathology, such as cerebrospinal fluid biomarkers is still unclear (64, 65), and further studies are needed on this topic.

## Cognitive Intervention

Cognitive intervention is currently the NPT that has been better explored in dementia and represents the most robust alternative and/or complement to pharmacological treatment. Cognitive intervention is typically classified as cognitive stimulation (CS), cognitive training (CT), and cognitive rehabilitation (CR) (66). CS refers to a broad range of activities (i.e., reality orientation therapy, reminiscence therapy) aimed to enhance the general cognitive and social functioning of the individual (67). While CS consists in a global approach to arouse all cognitive domains, CT focuses on a particular cognitive function (e.g., attention, memory, executive functions, language) through a set of standard tasks to improve or maintain—as is the case for neurodegenerative diseases—its normal functioning as long as possible. CR refers to a tailor-made approach which sets realistic goals to help patients and their families in everyday life. To achieve these aims, the CR therapist may choose a restorative or a compensative approach (68).

Even though the distinction between CS, CT, and CR is clear, the literature on cognitive interventions is, to some extent, confused. These terms are often used interchangeably, even if they refer to distinct approaches with a different rationale. Moreover, CR has been rarely used in dementia, because it may be difficult to apply in this condition. For these reasons, our review will focus on studies exploring CS or CT for people with dementia.

CS is currently the intervention with the most robust evidence. Several studies reported an improvement of general cognitive functioning in patients with mild-to-moderate dementia after CS sessions of variable length (69–74). A recent meta-analysis showed that CS has a moderate effect size on the Mini Mental State Examination (75) and a small effect size on the Alzheimer's Disease Assessment Scale-cognitive subscale (76), which represent two commonly-used general cognitive functioning outcomes, in patients with dementia (73). There are still few data on the efficacy of CS on other outcomes, such as mood, behavioral symptoms, QoL, and well-being. However, preliminary results indicate that CS may improve QoL and well-being of caregivers of people with AD and dementia (69, 70, 74).

The evidence on CT for people with dementia is less robust. A recent systematic review indicated that CT may improve patient's performance in trained tasks and similar exercises but has no consistent effect on everyday functioning (77). Most RCTs showed no effect of CT on other outcomes, but their low quality impede any solid conclusion (70–72, 74, 77).

Some studies documented the association of CS and CT did not result in better outcomes than single interventions (73, 74).

The Cognitive Stimulation Therapy (CST) was proposed as a structured and methodologically sound CS protocol (78, 79). CST is currently considered the evidence-based NPT of choice for the cognitive symptoms of mild-to-moderate dementia of any etiology, including vascular one (80–83). Moreover, CST is at present, the only one for which international RCTs consistently demonstrated significant improvements to cognition and QoL, using the same CST program which has been culturally adapted using standardized guidelines. CST involves 14 or more sessions of themed activities, usually related to tasks including money use, word games, word association, object categorization, current affairs and famous faces, and is typically run twice weekly (78). Each CST session is aimed to stimulate cognitive abilities and social relationships with other members and operators (78, 79). Several studies suggested the area of major CST efficacy in people with mild-to-moderate dementia is general cognitive functioning, but some cognitive areas, namely memory, orientation and language comprehension seems to be those with larger improvement following CST in an open study (84).

Several issues (e.g., health or mobility problems, services not offering this treatment) could undermine the participation of patients with dementia to CST groups (85) and the interest for individualized CST (iCST) is increasingly growing (85, 86). However, a recent RCT showed that a home-based iCST program had neither effect on cognition and QoL of patients with mild-to-moderate dementia, nor on QoL of their caregivers (87). The reasons for this negative finding might include the lack of additional stimulation from participation in a group social context and the difficulty for the caregiver to administer the iCST program in the proper way, being not professional, and because of the difficult relationship with the person with dementia.

In conclusion, while the CST program—a precise technique with a specific manual and training—has a robust evidence-based body of data, the high heterogeneity in terms of duration of treatment, length, and number of single sessions and severity of dementia impede any definite conclusion on the efficacy of other CS and CT interventions (73).

## Non-pharmacological Management of Behavioral and Psychological Symptoms of Dementia

Behavioral and psychological symptoms of dementia, i.e., the so-called BPSD, are characterized by alterations of perception (hallucinations, misidentification), thinking (delusions), mood (depression, anxiety, apathy), and behavior (aggression, agitation, disinhibition, wandering, socially or sexually inappropriate behavior) (88), being highly prevalent in all types of dementia, i.e., 50–80% in AD, >80% in fronto-temporal dementia, 50–70% in Lewy body dementia and vascular dementia (89, 90). BPSD may occur in all stages of AD, and even in people with MCI, but there is no agreement on the possible relationship with disease evolution. Indeed, although some studies showed that the prevalence and severity of BPSD increase with dementia severity, other reports suggest they are more frequent in the moderate AD stages, and diminish with disease progression (89, 90). Factors that may influence BPSD occurrence include premorbid personality, younger age, poor education, depression, high burden, concurrent medical conditions, and drugs. Environmental factors such as noise, overstimulation, isolation, inadequate temperature, change of routine, as well as inappropriate relationship between patient and caregiver (e.g., communication style, body language) or the inability of the patient to express his needs, may all trigger BPSD (91).

BPSD represent the most frequent cause of institutionalization and drug prescription, increase patient's disability, worsen patient's and caregiver's QoL, contribute to increase costs, but their influence on cognition is unclear (92).

According to consensus papers based on expert opinion and systematic revision of RCTs, the general principles for BPSD management include the following steps: symptoms evaluation (i.e., type, entity, frequency), recognition and treatment of possible organic causes or triggering factors, NPT, pharmacological therapy, educational, and psychological support for the caregiver (93–96).

Considering the limited evidence and concerns on the safety of drug treatment (94–99), all guidelines agree that individualized and person-centered NPT are the first-line treatment for BPDS and the use of a pharmacological strategy should be considered only later, using the least harmful drug for the shortest time.

Some theoretical models have been proposed to explain the role of non-pharmacological approaches to BPSD (100). The progressively lowered stress threshold (PLST) model suggests that, with disease progression, people with dementia experience increasing vulnerability to stress and external stimuli because of the environmental demand. According to the PLST model, minimizing environmental demands that exceed functional capacity, and regulating activity and stimulation levels throughout the day can reduce agitation. According to the competence-environmental press (CEP) model, the highest individual functioning results from optimal combinations of environmental conditions and personal competencies. The CEP model suggests that the right balance between individual ability and external environmental demands results in adaptive behaviors, while excessive or reduced environmental activities may result in BPSD. Both models suggest that BPSD can be reduced or managed by modifying environmental factors that place too much demand or pressure on the patient.

A different approach is provided by the need-driven, dementia-compromised behavior (NDB) that conceptualized BPSD as the result of the combination between the inability of the caregiver to understand patient's needs and the inability of the patient to express them. Therefore, BPSD may represent patient's attempts to express physical or emotional distress due to unmet needs. According to this model, NDB are induced by both background (i.e., cognitive, neurological, health, and psychosocial variables) and proximal factors (i.e., environmental and situational issues such as pain, fatigue, and noise); while background factors are quite stable, proximal ones are modifiable and represent the target for the interventions (101, 102).

The number of reports and reviews on the efficacy of NPT of BPSD has increased in recent years. Most studies have been performed in nursing homes on patients with advanced dementia. The interventions range from sensorial to psychological and behavioral approaches, and include environmental redesign, validation therapy, reminiscence therapy, light therapy, aromatherapy, massage and other sensory-based strategies, acupuncture, music therapy, pleasant events and activity engagement, behavioral management techniques, caregiver training (70, 99, 103–107). Potential benefits of these interventions have been documented, but the strength of evidence is overall low and the great heterogeneity in terms of type of intervention, follow-up duration, type of outcome measures hamper comparison across studies. In particular, there are no conclusive results for validation and reminiscence therapy, the effectiveness of light therapy is not clear, aromatherapy is better than no treatment. The small body of evidence for other sensory interventions, such as massage and touch therapy, suggests they could be viable treatment options, especially given the ease of implementation and minimal training involved. No effect has been documented for acupuncture and transcutaneous electrical nerve stimulation. Music therapy and behavioral management techniques seem to cause a short-term reduction of BPSD, but long-term efficacy is unclear.

In general, no conclusions can be drawn on the safety of these NPT, because this feature is largely unexplored (103). Some reports indicate the NPT to BPSD to be potentially cost-effective (70, 99, 106), but further research is needed on this point.

Factors that may predict a better response to these interventions include a higher level of cognitive functioning, fewer difficulties in ADL and communication, and relative preservation of speech, while staff barriers and pain are associated to worse outcome (108).

Independently from the results, all studies underline the relevance of a tailored individualized intervention and a careful and close monitoring of the outcomes to ameliorate and modulate the treatment (109, 110). Since pain and discomfort may cause difficulties in communication and self-management, and contribute to BPSD, these factors should be carefully searched and treated (111, 112).

In our opinion, the type of setting of interventions is an important point, as it may influence aims of the treatment and outcomes. The majority of studies on BPSD have been performed in nursing homes, where different health professionals (i.e., physicians, psychologists, nurses) are involved, and they may interact to offer a better outcome (111). In contrast, as caregiver is often the only figure that manage patients' BPSD at home, future studies should address the role of NPT for BPSD in non-institutionalized people with dementia (106).

## Occupational Therapy

Disease progression reduces the ability to participate in occupations—mainly ADL, IADL, leisure and social activities—in patients with dementia, leading to a negative impact on QoL and well-being of patients and caregivers (113–117). Therefore, improving and preserving ADLs is one of the most important patient-related outcome in dementia (118).

To increase functional abilities and enhance independence, occupational therapy (OT) uses a combined approach including activity simplification, environmental modification, adaptive aids, problem-solving strategies, skill training, and caregiver education (119, 120), according to the bio-psycho-social model of health proposed by the ICF (116). An RCT on patients with mild-to-moderate dementia living in the community showed that a multimodal OT approach, including cognitive and behavioral interventions to train patients to compensate for cognitive impairment and caregivers to cope with patients' abnormal behavior, was effective in improving ADL and in reducing caregiver's burden with effects lasting for up to 12 weeks (121). A review including seven RCTs showed that OT interventions may maintain physical function and ADL in community-dwelling persons with dementia (105, 122).

OT seems also effective in reducing BPSD, increasing patient's participation, improving physical performance, and QoL and reducing negative communication (123–129). These positive results have been confirmed in a recent review that showed OT interventions based on sensory stimulation, environmental modification and functionally oriented tasks, to be effective in improving BPSD and depression in dementia (130). Among environment-based interventions, ambient music, aromatherapy, and Snoezelen (i.e., a soothing and stimulating multisensory environment) were modestly effective in reducing agitation, but they did not have long-term effects, while the evidence to support bright light therapy for mood and sleep disorders was only preliminary (131).

An evidence-based review suggested that patient-centered leisure activities that involve social interaction may enhance caregiver satisfaction with visits for residential patients, while interventions focused on ADL and IADL may ameliorate patients' well-being and QoL. Social participation interventions, mainly involving people in the early or middle stage of the disease when verbal competence is still fairly spared, were shown to have at least short-term positive effect on patient's well-being (132).

Gitlin and collaborators proposed a 4-month structured home occupational therapy intervention, the Tailored Activity Program (TAP), that provided persons with dementia with activities tailored to their abilities, while caregivers were trained to effectively use these activities in daily life and to generalize them to different settings (133). Besides being effective in reducing caregiver burden, in that caregiver had significantly more free time, TAP was demonstrated to be highly cost-effective (123).

Some general considerations have been issued on OT: (a) programs should be tailored to elicit the patient's highest level of retained skill and interest; (b) cues for assisting people with AD to complete tasks should be short and provide clear direction; (c) compensatory strategies in the form of environmental modifications and simple adaptive equipment should be specifically tailored to the specific needs of single patients; (d) caregiver training and involvement are essential in implementing individualized programs (134).

OT is recommended by several guidelines for dementia management (135–137). The recently published clinical practice guidelines and principles of care for people with dementia in Australia reported important evidence-based recommendations for occupational therapists, based on the most updated high-quality evidence (138). However, well designed RCTs are lacking on this topic and often the studies use different approach, lacking specificity. Therefore, more research is needed to identify specific OT components and the optimal dosage of the intervention and to evaluate the long-term effects and the contraindications of OT in patients with dementia.

Subtle functional changes in highly cognitively demanding activities have been reported in individuals in the early stage of the disease, suggesting that, similarly to cognition, function declines along a continuum from healthy aging to dementia (139–141). Incorporating more challenging and high-level activities into the assessment of patients with early dementia may increase the sensitivity of functional measures, thus enabling early detection of impairment and expanding the window for timely OT interventions (142). These points may represent directions for future research on this topic.

## Psychological Therapy

In recent years, the growing awareness of the deep emotional impact of the diagnosis of dementia and of the need of a holistic and individualized approach to the disease, has led to a rising interest on the psychological care for persons with dementia (143).

Actually, experiencing depression and anxiety is very common in people with dementia, even if there is a lack of consensus on their prevalence rates as diagnostic criteria may vary among studies (144–147). All studies agree that depression and anxiety may have significant functional impact. Consistent evidence suggests that, in people with dementia, the severity of depression increases the severity of neurological impairment, institutionalization (148, 149), and caregiver burden (150), while anxiety is associated with decreased independence and increased risk of nursing home placement (151).

Although different from each other, depression often co-occurs with apathy. Apathy, which is defined as a loss or diminution of goal-directed behavior, cognition or emotion (152) is one the of the most common neuropsychiatric symptom in AD, with a prevalence ranging from 19 to 88% (153). Even if symptoms like social withdrawal or reduced initiation and motivation may occur both in apathy and depression, true apathy is usually not associated with depressive symptoms, such as sadness, guilt, or hopelessness (154). The presence of apathy seems to be related to greater caregiver burden, faster functional impairment, reduced QoL and increased morbidity, being a reliable longitudinal predictor of death in people with dementia (155–157).

Many recommendations underscored that the treatment of anxiety and depressive symptoms should be an essential part of the management of AD and other dementias (93, 152, 153) and suggested that psychological interventions could be effective for these patients (158). However, empirical evidence supporting the efficacy of psychological interventions is rather sparse (159), mainly because a broad group of interventions that are termed as psychological ones have been included in reviews (160, 161), but actually are not based on well-defined theory-driven psychological interventions.

According to the World Health Organization, cognitive behavioral therapy, psychodynamic therapy, interpersonal therapy, and supportive counseling (i.e., Rogersian person-centered therapy) are the main psychotherapeutic approaches with evidence of effectiveness in treating depression and anxiety in adults (162). Studies that have adopted one of these approaches have been included in a recent systematic review on psychological interventions for anxiety and depression in dementia or MCI (six RCTs, psychological treatment: *n* = 216 patients, usual care or placebo intervention: *n* = 223) (163). The Authors concluded that there is evidence that psychological interventions added to usual care can reduce symptoms of depression and clinician-rated anxiety in dementia and that psychological interventions have the potential to improve patient's well-being, while no effect was found on ADL, self- and caregiver-rated patient's QoL, BPSD, cognition, or caregivers' self-reported depressive symptoms (163).

A recent systematic review concluded that the strongest evidence supported the use of short-term group therapy soon after dementia diagnosis to reduce levels of depression and enhance QoL, while tailored and multi-component interventions seemed to reduce inappropriate behaviors in patients living in nursing homes with mild-to-moderate levels of impairment (164).

Systematic reviews indicated a variety of NPTs (e.g., one-to-one activities, activity programs tailored on patients' preferences, individualized cognitive rehabilitation, music therapy, art therapy) as potentially effective and safe in reducing apathy, although none of them is strictly a psychological intervention (165, 166).

Overall, the proposed psychological approaches were methodologically heterogeneous, some of them lacked specificity and conceptual clarity and often the description of the interventions was quite generic. Moreover, most studies included patients in the first stage of dementia, because cognitive decline prevents the understanding and feasibility of psychological therapy in advanced stages.

In conclusion, the current evidence for psychological support and psychotherapy in AD and dementia is limited and not conclusive. Therefore, further studies are needed to demonstrate which psychological interventions may lead to better outcome and to identify common factors across different approaches that may be effective in enhancing well-being of persons with dementia.

## Multicomponent and Multidimensional Strategies

The proposal of multidimensional protocols of treatment is based on the hypothesis that combining different approaches may be the most suitable intervention to improve different outcomes in AD. An Italian group developed the Multidimensional Stimulation Therapy (MST), a multidimensional approach that combined cognitive stimulation, recreational activities, OT and physical exercises, and demonstrated that MST was more effective than a cognitive-specific intervention in improving both behavioral and functional outcomes in AD patients (167, 168). A recent fMRI study suggested restoration of neural functioning as the mechanism underlying the positive effects of MST (169).

Similarly, the Meeting Centers Support Program (MCSP), a supportive person-centered approach for mild-to-moderate dementia patients living in the community and their caregivers was implemented in the Netherlands. MCSP is based on the combination of recreational activities and psychomotor therapy for patients, psico-educational and support groups for caregivers, social activities for both, and regular center meetings (170). MCSP yielded promising results in terms of user satisfaction, reduction of patients' behavioral and emotional disturbances, decrease of caregivers' burden and delayed institutionalization (170–173). A study exploring the dissemination and implementation of MCSP to different European countries is currently ongoing (174).

Actually, some systematic reviews demonstrated that multicomponent interventions were more effective than single activities in improving general well-being of both persons with dementia and their caregivers (70, 175), and in hospitalized older adults with cognitive impairment (176, 177).

As dementia represents a complex disease with cognitive, psychological, functional and behavioral symptoms, the adoption of multicomponent approaches may represent a winning strategy, in agreement with the biopsychosocial model of care, upon which any rehabilitative intervention is founded. The encouraging results obtained so far suggest further studies in this area of research.

## Complementary and Alternative Medicine

Complementary and alternative medicine (CAM) has become more commonly used over the last decade and has been applied to a wide range of health problems, including dementia. We will report evidence on the main CAM types, i.e., aromatherapy, music therapy, art therapy, massage, and touch. These NPTs are part of the main category of sensorial stimulation techniques, i.e., interventions aimed to promote the arousal of the patients' senses (178).

## Aromatherapy

Among CAM, aromatherapy is probably the most familiar to consumers. Aromatherapy is part of phytotherapy and is based on the use of pure essential oils from fragrant plants to relieve health problems and improve QoL. The potential effects of essential oils are varied, including promotion of relaxation and sleep, relief of pain (179), reduction of depressive symptoms. Aromatherapy might be a useful intervention for people with cognitive disturbances, who are confused, or for whom verbal interaction is difficult and current conventional medicine provides only partial benefit (180). Indeed, aromatherapy has been used to address BPSD (181–186) and sleep disturbances (187–189) and to stimulate motivational behavior (190). A meta-analysis identified only seven RCTs and concluded that the benefits of aromatherapy for people with dementia are equivocal, mainly because of severe methodological problems (191). Therefore, the effects of aromatherapy in this condition should be better explored in high-quality RCTs.

## Music Therapy

Music therapy (MT) can be defined as an NPT that uses music or sound as a non-verbal communication tool to induce educational, rehabilitative or therapeutic effects in several disease, including dementia. Music-based interventions can be conducted in single patients or in group, and they most consist of singing, listening, improvising or playing along on musical instruments (192). We can therefore classify MT in active and passive interventions. Since most studies on MT included both active and passive techniques, which type of intervention can be more effective in dementia is still unclear (193).

There is substantive literature reporting the importance and benefits of music and music therapy programs for older adults, and more specifically for those with dementia (194–199). Neurophysiologic and psychological data provide the theoretical basis for the use of music therapy. Studies showed that sound can exert a significant action on the brain (200–204) by activating a large number of cortical and subcortical areas, and, particularly, the limbic and paralimbic areas devoted to emotional processing (205). From a psychological point of view, music facilitates communicative and relational processes and emotional expression (206). Moreover, since music can stimulate both motor and cognitive functions, it can be an efficient tool for rehabilitation (207). A growing amount of evidence shows that patients with dementia can enjoy music, and their ability to respond to music is potentially preserved, even in the late to severe stages of disease when verbal communication may have ceased. Several studies reported the efficacy of music to treat BPSD (208–213), to enhance communications, relational processes and participation (199, 214–216), and to maintain or increase cognitive functions (217–222). However, reviews on this topic pointed out the methodological limitations and the scarce quality of previous studies and questioned the specificity of the effect of music that was found to be no more beneficial than other pleasant activities (223, 224).

A recent meta-analysis concluded that a music-based therapeutic intervention probably reduces depressive symptoms but has little or no effect on agitation or aggression, emotional well-being or QoL, overall behavioral problems and cognition in dementia, while effects on anxiety or social behavior, and long-term effects are unclear (192).

A recent RCT found that music therapy added to pharmacotherapy yielded no further benefits for language and verbal communication in comparison to pharmacotherapy alone, but this integrated approach could improve the psycho-behavioral profile (225).

In conclusion, the question whether music may have specific effects in patients with dementia is still open. Future studies should employ larger sample sizes, include all important outcomes, in particular positive ones, such as emotional and social well-being, and examine the duration of the effect in relation to the overall duration of the treatment and the number of sessions (192, 226).

## Art Therapy

The British Association of Art Therapists defines art therapy as “a form of psychotherapy that uses art media as its primary mode of communication” and underscored that (a) clients who are referred to an art therapist need not have experience or skill in art; (b) the art therapist is not primarily concerned with making an aesthetic or diagnostic assessment of the client's image; (c) the overall aim of its practitioners is to enable a client to change and grow on a personal level through the use of art materials in a safe and facilitating environment” (227).

Whether art therapy can be useful and may represent an effective rehabilitative strategy for people suffering from dementia is a debated issue (228, 110). It has been suggested that patients with dementia can produce and appreciate visual art and that aesthetic preference can remain stable, despite cognitive decline (229).

Case reports, observational studies, small trials and anecdotal evidence suggest that art therapy can engage attention, provide pleasure, and improve BPSD, social behavior, and self-esteem, contributing to the individual sense of well-being in dementia, and that these effects may be long-lasting (230–243). However, few high-quality studies have been performed so far on this topic, and there is too little empirical evidence to prove the effectiveness of this type of CAM. Therefore, RCTs with adequate methods and appropriate outcomes are needed to support the efficacy and define optimal conditions for the use of art therapy in dementia.

## Massage and Touch

Massage and touch have been suggested as a CAM or supplement to other treatments to reduce a range of conditions associated with dementia. Evidence suggests that massage and touch may relax older people with dementia, reduce some BPSD, such as pacing, wandering and resisting care, improve appetite, sleep and communication problems, and, to some extent, counteract cognitive decline (244–250).

Massage seems to produce different physiological effects such as a decrease in pulse and respiration and an increase in body temperature (251). Physiological models suggest that the effects of massage may be mediated by the production of oxytocin that can reduce discomfort, agitation, blood pressure and heart rate, and improve mood. On the other hand, psychological models suggest that massage can help people with dementia to retain a sense of meaningful and reassuring communication even when words fail and may help to activate memories (252).

A recent meta-analysis of 11 RCTs showed that massage and touch interventions may have significant effect on physical non-aggressive, verbal aggressive and non-aggressive behavior, but the relatively small sample size and the low quality of the included studies do not allow to draw a firm conclusion on the effect on BPSD or any useful suggestion for clinical practice (253).

As for other CAM types, RCTs of better methodological quality are needed to provide evidence supporting the benefit of massage and touch in dementia.

## New Technologies

The availability of new technologies, their diffusion and, for some of them, the relatively cheap price, resulted in a progressively larger number of studies exploring their role in dementia. We will briefly summarize evidence on the application of information and communication technologies (ICT), assistive devices, domotics, virtual reality, gaming and telemedicine to monitor, and treat people with dementia.

## ICT, Assistive Devices and Domotics

Recent technological advances in ICT, including computational devices, robotics, machine learning algorithms, miniaturization of sensors and technologies, as well as the reduced costs of these devices increased the availability of intelligent devices and technology-embedded environments to support the daily life of persons with dementia and their caregivers, and to assess disease severity and progression (254–256). Nowadays, devices and sensors can be integrated into the patient's environment as part of a “smart” or automated home, or in the patient's clothes to offer healthcare professionals a much clearer view of the patient's condition and activity than that available from short periods of monitoring in a clinical setting (257). In particular, ICT allows continuous monitoring of patient's performance and actions in real time and real-life situations (258–260).

Moving freely outdoors is considered an important ADL outcome associated with the ability to maintain independence and participation. The development of technologies, which allow people with dementia to stay outdoors in a safe and secure way, is considered a priority research area. Therefore, tracking technologies that record the position of the patient based on global positioning system (GPS), and can localize him/her if lost is the most developed area of ICT research in dementia (261–265). Tracking technology devices can promote perceived safety and security among caregivers and relatives of people with dementia (266), in that they can locate the patient at any moment and represent a precious support in everyday life (263, 267). Many studies in this area focused on the perspective of relatives and/or healthcare staff, but only a limited number of them explored the experience of people when wearing a GPS tracking technology (259, 262, 268, 269).

Another application of ICT is represented by devices that can be used for surveillance and management of high-risk behaviors and may replace more severe forms of physical restraints (270–272). Acoustic sensors placed in the living room or bedroom can record noise, or patient's movement. Cameras or GPS devices can also be used to allow caregivers or staff to take actions when the patient is in a potentially dangerous condition, without restricting freedom of movement. Furthermore, patients can wear a chip in their clothes to give them access to certain parts of the building, while preventing them to move to other forbidden or dangerous areas (266).

However, the application of surveillance technologies to the care of people with dementia led to considerable ethical debate. Indeed, opponents of this approach claim that the continuous monitoring may represent a breach of patient's privacy and freedom and argue that tracking devices may diminish human contact between patients and the environment (273). A systematic review on the ethical and practical concerns of surveillance technologies in residential care for people with dementia concluded that there is no consensus and underlined the need for clearer policies on this topic (274). A recent descriptive review showed that a significant portion of current intelligent assistive technology is designed in the absence of explicit ethical considerations, and this may represent a structural limitation in the translation from designing labs to bedside (275). The Authors called for a coordinated effort to proactively incorporate ethical considerations early in the design and development of new ICT products (275).

ICT has also been applied to monitoring and prevention of falls in dementia. Body-worn gyroscopes measuring angular velocity, sensors to measure the acceleration of the trunk, and inertial sensors may detect when a fall occurs, along with the causes of the fall (276–279). Among non-wearable systems cameras, motion sensors, microphones and floor sensors have been applied to this aim (280, 281). Information obtained by accelerometers contained in a wristband may result in vibratory and visual feedback to increase the patient's awareness of their risk of falling (282). Similarly, a shoe insole that delivers vibration may prevent falls and improve gait and balance. Research projects are currently exploring how exoskeletons can correct and assist postural balance and improve overall function. Technology will hopefully provide many more options for assessment, prevention, and intervention in this area of health care (283).

Video and audio analysis techniques, computerized testing and actigraphy, besides being useful to promote patients' safety, may represent promising tools to improve functional and cognitive assessment of patients with AD (256). For instance, automatic speech analysis techniques through computerized speech recognition interfaces could represent a non-invasive and cheap method to collect information on verbal communication impairment in patients with cognitive disorders (284). Video processing technologies can be adopted to obtain pragmatic, ecological, and objective measures to improve assessment of ADL, and to provide additional information that cannot be gathered in a clinical setting (285).

ICT can be used for the assessment of agitation, one of the most challenging symptoms to manage for caregivers (286, 287). A core feature of agitation is excessive motor activity, and accelerometers can objectively measure abnormal motor activity, speech analyses may assess verbal aggression, and automated video analysis and activity recognition techniques can detect activities and movement sequences that underline physical aggression (288). Sensors measuring electrodermal activity could early detect agitation in persons with dementia. These devices could theoretically support nursing staff in recognizing early warning signs of BPSD, but further studies in a clinical setting are needed to confirm this hypothesis (289).

Overall, this is a challenging domain for clinicians, who should define the clinically relevant markers, and for ICT engineers, who should adapt the technical constraints to the clinical requirements.

Assistive technologies can support and facilitate disabled people in ADL (290). For instance, external cueing systems can assist people with cognitive disabilities by reminding them to perform a task at the appropriate time (e.g., take their pills), or by providing guidance through a task (291–293), and sensors recording a patient's position in space may help in navigation through environment (294). Moreover, assistive technologies may help to support decision-making in vocational and personal health domains (295), to read (296), or use the telephone (297).

Domotics is another important field of research, because nearly three quarters of older adults with dementia are cared for in their own homes (298), and there is a strong correlation between home environment and disability-related outcomes (299). Dementia-specific adapted home environment and related assistive devices may be helpful for both the patient and the caregiver (300–302). Changes to home environment may result in lack of familiarity for the patient, and earlier home modifications might enable individuals with dementia to adjust to their adapted environment (303).

Future smart homes will incorporate intelligent interfaces embedded in everyday objects, such as furniture, clothes, vehicles, and smart materials to observe the residents and provide proactive services (304). One of the key-supporting features of a smart home is the ability to monitor the ADL and safety of people, and to detect changes in their daily routines. With the availability of inexpensive low-power sensors, radios, and embedded processors, smart homes may be equipped with large amounts of networked sensors, which collaboratively process and make deductions from the acquired data on the state of the home, and the activity and behavior of its residents (305–310). Therefore, the smart home technology seems to be a promising and cost-effective way of improving home care for people with dementia in a non-obtrusive way, allowing greater independence, maintaining good health, preventing social isolation, and relieving the workload from family caregivers and health providers (311).

The data on domotics are encouraging but some factors, e.g., the accessibility and affordability of these devices and the limited caregiver's awareness of their availability and utility, may prevent a more widespread use of these technologies (312).

In conclusion, ICT applications are rapidly expanding to support assistive and clinical tasks in people with dementia, but structural limitations to successful adoption include partial lack of clinical validation and insufficient focus on patient's needs. Clinicians and stakeholders involved in the implementation and management of dementia care across ICT applications should collaborate to facilitate the translation of medical engineering research into clinical practice (313).

## Virtual Reality and Gaming

Virtual reality (VR) allows a user to navigate through, and interact with, a virtual environment that is a 3D digital space generated by computing technology and consisting of objects or entities seemingly “real” because they share at least one attribute of the real thing, without sharing all of its physical characteristics (e.g., volume, weight, surface friction) (314). The potential applications of VR systems to assess and train AD patients has been reported by several studies (315–320). Cognitively impaired patients can be exposed to computer-generated virtual environments, where they can safely perform quasi-naturalistic real-life activities and tasks to assess and/or treat cognitive and motor deficits under a range of conditions that are not easily controllable and quantifiable in the real world (321). The most specific domains for VR diagnostic and training purposes in AD patients include attention (322), executive functions (323–326), memory (327–333), orientation (322, 327, 334–336), and ADL (337, 338). Research is currently focusing on the design of VR based tests aimed to be more ecological and sensitive compared to common pen-and-paper tests. A recent study proposed a novel AD screening tests based on virtual environment and game principles to evaluate memory loss for common objects and recent events, and language changes (339).

The advantages of VR in comparison to traditional assessment and training tools are represented by the naturalistic situations, the safety from potential dangers that could occur in real-life situations and the enhancement of motivation because of its gaming, interactive, and immersive quality. For these reasons, computer-driven simulations of real-life environments can bridge the gap between traditional test outcomes and compensatory strategies that are transferable to daily life.

Since most of the evidence on the role of VR come from single case reports or to restricted samples, the debate on its role in patients with dementia is still open.

In this scenario, serious games (SG), i.e., interactive virtual simulations to represent real situations, otherwise hardly reproducible, allow the player to act in a real-life-like environment, or a fictitious scenario that serves as a gym for deliberately decontextualized learning. SG are widely recognized as promising non-pharmacological tools to assess patient's cognition and function (340, 341), and to treat, stimulate, and improve patient's well-being (342). Boosted by the publication of a Nature letter showing that video game training can enhance cognitive control in older adults (343), SG specifically adapted to people with AD have been developed and employed to train physical and cognitive abilities, showing preliminary but encouraging results (341, 344–348).

A recent literature review concluded that game-based interventions can positively affect both physical health by improving balance, gait and voluntary motor control and cognitive function by enhancing attention, memory, and visuo-spatial abilities in older adults and in patients with dementia (349). Both physical and cognitive games seem to have a positive impact on social and emotional function, improving mood and increasing positive affect and sociability (350).

Since the use of SG in neurodegenerative disorders is expanding, recent studies have provided recommendations in terms of ergonomic choices for their design, and to create customize SG specific to the target users (351). Considering that the actual recommendations are gathered from a relatively small group of experts working in the domain of SG for health, further work is needed to verify if they hold true for a wider expert population, and their applicability to patients.

On the basis of these preliminary data, clear evidence on the effectiveness of VR and SG for the assessment and training of people with AD requires further research that should adopt methodologies based upon best practices from clinical trials and incorporate behavioral and neurophysiological data.

## Telemedicine

Technology provides new opportunities for interventions to improve quality and access to health care. Since dementia is a chronic disease, with issues related to continuous long-term care and limited accessibility to medical service, telemedicine could be a valuable mean to provide assessment and care to dementia patients at home (352). Indeed, telemedicine can provide follow-up care to help people with dementia to maintain their independence and continue living in their own homes, improve their safety, (353, 354), ameliorate clinical outcomes, and reduce access to health services (355, 356). Moreover, likely because of the reduction of the discomfort due to travel, telemedicine was found to be an independent predictor for increased treatment duration (357). Telemedicine appears to be very promising and effective in the context of dementia assessment, especially in countries, such as the USA, where there are several rural retirement communities that are still medically underserved. A recent study conducted in a Southern California community showed not only that telemedicine was able to improve access and quality of dementia care, but also that most of the patients preferred the telemedicine consult compared to the traditional one (358).

Several studies confirmed the feasibility to perform cognitive assessment via telemedicine in elderly subjects with dementia and showed a good agreement with conventional face-to-face testing, and a high level of user satisfaction (359–362). However, since data are still preliminary, there is no consensus on the best practices for conducting neuropsychological assessment by telemedicine (362).

Besides the diagnostic evaluation and follow-up monitoring, telemedicine interventions for dementia include internet-based information and support groups, robotic companions, the use of smartphones to report symptoms (258, 363, 364), and cognitive rehabilitation training (365).

Telemedicine has been applied to support caregivers of people with dementia through in-home video monitoring and recording and feedback to help managing difficult situations (366). Telephone-based support groups is a promising way to provide support for caregivers, but research is only at the beginning (367). A systematic review on technology-driven telemedicine interventions, such as online training and support programs, for caregivers of persons with dementia concluded that included studies yielded positive findings in terms of mental health outcomes, but their marked heterogeneity impeded robust conclusions (368).

Overall, establishing systems for the care of dementia patients and their caregivers using telemedicine technologies seems to be feasible, but the evidence on clinical benefits is still scarce, cost-effectiveness data are lacking, and there is very little evidence of widespread practical applications (369).

## Limitations of the Study

The main limitation of the present review is its narrative design, but a systematic one would have not been feasible because of the larger number of studies on NPT in AD and dementia. Another limitation is the use of two search engines, only (i.e., PubMed and the Cochrane database of systematic reviews). Searching in other databases (e.g., Embase, PsycINFO, Web of Science) would have yielded more pieces of literature, but it has been suggested that MEDLINE, which is completely covered by PubMed, may produce a sufficiently extensive coverage (370). A study comparing 15 databases for RCTs on CAM showed low overlap between databases and indicated that comprehensive CAM literature searches will require multiple databases (371) but this appear a minor problem in the present review because of the overall low quality of studies on CAM interventions.

## Conclusions

Appropriate management of patients with AD and dementia is a significant public health concern, given the limited effectiveness of pharmacological therapies combined with their potentially life-threatening side effects. The development of effective NPT for these conditions is of paramount importance, and a large number of interventions has been proposed.

The interventions reviewed in this paper show different level of evidence of efficacy on different outcomes that are summarized in Table 1. Some of them share methodological problems that are common to all non-pharmacological studies, which are typically practice-oriented. They include small number of high-quality studies or double-blind RCTs, small sample sizes, heterogeneity in terms of study design, type of intervention and outcomes, uncertainty about the clinical significance of outcomes. The landscape of clinical trials has changed since the introduction of randomization 80 years ago, and new challenges in their design, conduction, and interpretation have emerged. Evidence-grading systems are biased toward RCTs, and they may lead to understatement of findings from non-RCT studies (372). The direct transposition of the traditional methods of drug RCTs to the rehabilitation and NPT scenarios raises some issues, and innovative trial designs would be helpful in these settings. Moreover, cost-effectiveness studies are needed to better address the role of NPT for dementia.

**Table 1:** Summary of the level of evidence, main positive outcomes, and limitations of the non-pharmacological treatment for Alzheimer's disease and dementia.

**Treatment**	**Subtype**	**Evidence**	**Main positive outcomes**	**Limitations**
Exercise and motor rehabilitation		Moderate	•Reduction of BPSD •Improvement of ADL	•Limited evidence for cognition, QoL, depression, mortality, caregiver burden •Few comparative studies exploring the most effective exercise intervention •Unclear duration and intensity of activity
Cognitive intervention	CS	Moderate	•Improvement of general cognitive function •Small effect on mood and QoL of patients and caregivers	•Unclear long-term effect on cognition •Heterogeneity of the outcomes
	CT	Small	•Enhancement of performance in trained and similar tasks	•No generalization effect to daily function •Low quality studies
	CS+CT	Small	•Improvement of general cognitive function	•Heterogeneity of treatment •Heterogeneity of dementia severity •Few high quality RCTs
	CST	Moderate	•Improvements of general cognitive function and specific cognitive domains •Small effect on QoL •Improvement of caregiving relationship •Small effect on depressive symptoms and QoL of caregivers	•Not all services offer this treatment •Unclear effect on BPSD and ADL
Occupational therapy		Small	•Improvement of ADL, social participation, QoL •Reduction of BPSD and depression •Reduction of caregiver burden	•Unclear optimal duration •Unclear long-term effect •Mixed approaches •Few high quality RCTs
Psychological therapy		Small	•Reduction of depression, anxiety, and apathy •Enhancement of patient's well-being	•No comparative studies between different psychological treatments •No long-term studies •Few high quality RCTs
Multicomponent/multidimensional strategies		Small	•Reduction of BPSD •Improvement of some cognitive domains •Delayed institutionalization •Reduction of caregiver burden	•Persistence of effect •Generalization to everyday life •Limited implementation and dissemination •Few high quality RCTs
Aromatherapy		Very small	•Reduction of BPSD and sleep disturbances •Improvements in motivational behavior	•Severe methodological issues •Few high quality RCTs
Music therapy		Small	•Reduction of depression •Improvement in communication, social participation, relations and cognition	•Unclear long-term effect •Small sample size •Low-quality studies
Art therapy		Very small	•Reduction of BPSD •Improvement of attention •Improvement of social behavior and self-esteem	•Unclear long-term effect •No RCTs
Massage and touch		Very small	•Reduction of BPSD •Improvement of mood, sleep, appetite	•Small sample size •Low-quality studies

*ADL, activities of daily living; BPSD, behavioral and psychological symptoms of dementia; CS, cognitive stimulation; CST, Cognitive Stimulation Therapy; QoL, quality of life; RCT, randomized controlled trial; CT, cognitive stimulation*.

## Author Contributions

CZ, ES, and MB conceived and designed the study. All authors collected, analyzed, and interpreted the data. CZ, ES, AF, EM, SB, and MB drafted the manuscript. ST and RC revised critically the manuscript for important intellectual content. All authors approved the final version of the manuscript.

### Conflict of Interest Statement

The authors declare that the research was conducted in the absence of any commercial or financial relationships that could be construed as a potential conflict of interest.
